# Cigarette Smoke Toxins Deposited on Surfaces: Implications for Human Health

**DOI:** 10.1371/journal.pone.0086391

**Published:** 2014-01-29

**Authors:** Manuela Martins-Green, Neema Adhami, Michael Frankos, Mathew Valdez, Benjamin Goodwin, Julia Lyubovitsky, Sandeep Dhall, Monika Garcia, Ivie Egiebor, Bethanne Martinez, Harry W. Green, Christopher Havel, Lisa Yu, Sandy Liles, Georg Matt, Hugo Destaillats, Mohammed Sleiman, Laura A. Gundel, Neal Benowitz, Peyton Jacob, Melbourne Hovell, Jonathan P. Winickoff, Margarita Curras-Collazo

**Affiliations:** 1 Department of Cell Biology and Neuroscience, University of California, Riverside, Riverside, California, United States of America; 2 Department of Bioengineering, University of California, Riverside, Riverside, California, United States of America; 3 Graduate Division, University of California, Riverside, Riverside, California, United States of America; 4 Division of Clinical Pharmacology, University of California, San Francisco, San Francisco, California, United States of America; 5 Center for Behavioral Epidemiology & Community Health, School of Public Health, San Diego State University, San Diego, California, United States of America; 6 Department of Psychology, San Diego State University, San Diego, California, United States of America; 7 Indoor Environment Group, Lawrence Berkeley National Laboratory, Berkeley, California, United States of America; 8 MGH Center for Child & Adolescent Health Research and Policy, Harvard Medical School, Boston, Massachusetts, United States of America; The Ohio State University, United States of America

## Abstract

Cigarette smoking remains a significant health threat for smokers and nonsmokers alike. Secondhand smoke (SHS) is intrinsically more toxic than directly inhaled smoke. Recently, a new threat has been discovered – Thirdhand smoke (THS) – the accumulation of SHS on surfaces that ages with time, becoming progressively more toxic. THS is a potential health threat to children, spouses of smokers and workers in environments where smoking is or has been allowed. The goal of this study is to investigate the effects of THS on liver, lung, skin healing, and behavior, using an animal model exposed to THS under conditions that mimic exposure of humans. THS-exposed mice show alterations in multiple organ systems and excrete levels of NNAL (a tobacco-specific carcinogen biomarker) similar to those found in children exposed to SHS (and consequently to THS). In liver, THS leads to increased lipid levels and non-alcoholic fatty liver disease, a precursor to cirrhosis and cancer and a potential contributor to cardiovascular disease. In lung, THS stimulates excess collagen production and high levels of inflammatory cytokines, suggesting propensity for fibrosis with implications for inflammation-induced diseases such as chronic obstructive pulmonary disease and asthma. In wounded skin, healing in THS-exposed mice has many characteristics of the poor healing of surgical incisions observed in human smokers. Lastly, behavioral tests show that THS-exposed mice become hyperactive. The latter data, combined with emerging associated behavioral problems in children exposed to SHS/THS, suggest that, with prolonged exposure, they may be at significant risk for developing more severe neurological disorders. These results provide a basis for studies on the toxic effects of THS in humans and inform potential regulatory policies to prevent involuntary exposure to THS.

## Introduction

Despite efforts by governments and health organizations worldwide, cigarette smoking remains a serious health threat for smokers and nonsmokers alike [1]–[4]. Tobacco smoking causes significant human disease and economic burden worldwide, afflicting approximately 1.5 billion smokers while additional billions are at underappreciated health risk from exposure to cigarette smoke, with estimated annual costs of hundreds of billions of dollars. It has become clear that health threats are particularly serious for children who constitute a vulnerable population that cannot voluntarily avoid secondhand smoke (SHS) exposure. It is now well known that SHS is intrinsically more toxic than directly-inhaled firsthand smoke (FHS) [1], [2], [5], [6] and recently, a new and persistent potential threat has been discovered – thirdhand smoke (THS) – the accumulation of SHS on environmental surfaces that ages with time, becoming progressively more toxic [6]–[8].

The first complete ban in the world on indoor smoking in all public spaces (including bars and restaurants) occurred in 1990 in San Luis Obispo, CA. That legislation, and its expansion to many countries, was achieved only because of clear scientific evidence that SHS is dangerous to non-smokers. Now, more than 20 years later, evidence is emerging that THS exposure potentially poses similar health risks, especially for children. Several studies have affirmed THS as an underappreciated public health hazard [4]–[6], [9], [10]. Unfortunately, just as in the 1980s concerning SHS, today's public is skeptical about these risks [9]. Public convictions and support of THS exposure-control policies depend on biological evidence of THS toxicity.

Contamination of the homes of smokers by SHS residues (THS) is high, both on surfaces and in dust, including in children's bedrooms [11]. Re-emission of nicotine from contaminated indoor surfaces in these households can lead to nicotine exposure levels similar to that of smoking [12] and similar levels of contamination are found on surfaces and dust of vehicles of smokers [13], [14]. Recently, it was shown that THS remains in houses, apartments and hotel rooms after smokers move out [15], [16]. Tobacco-specific nitrosamines (TSNAs) are strong carcinogens present in the THS residues deposited on indoor surfaces. In addition, nicotine (and probably other tobacco components) adsorbed in large amounts (microgram per sq meter levels) onto surfaces can react with nitrous acid (HONO) to form TSNAs [7], [8], [17], [18]. Sources of indoor HONO and its precursors NO and NO_2_ include: a) smoking; b) combustion sources such as improperly ventilated gas stoves and heaters and c) infiltration of outdoor air pollution generated by vehicle exhaust or biomass burning [19], [20]. Thus, THS presents toxicants similar to those present in mainstream (MS) or SHS and, in addition, also contains new toxicants due to aging and reaction with other chemicals.

The exposure to tobacco smoke toxicants in THS can occur via ingestion, skin adsorption/absorption and/or inhalation [11]. In the US alone, nearly 88 million nonsmokers ages 3 and older live in homes where they are exposed to sufficient SHS+THS to produce significant blood levels of a tobacco-specific nitrosamine and cotinine (a metabolite of nicotine) [21].

Although the potential risks attributed to THS exposure are increasing, virtually nothing is known about the specific health implications of acute or cumulative exposure. Therefore, there is a critical need for animal experiments to evaluate biological effects of THS-exposure that will inform subsequent human epidemiological and clinical trials. Such studies can determine potential human health risks, design of clinical trials and potentially can contribute to policies that lead to reduction in both exposure and disease.

To address this need, we have conducted the first animal study on the effects of THS on several organ systems under conditions that simulate THS exposure of humans. We show that significant damage occurs in liver, lung and during healing of wounds. In addition, the mice display hyperactivity. The results of our study delineate the early stages of potentially serious THS-induced damage in each of these organs and in behavior, problems that are known to be associated in humans with THS and SHS but have not previously been identified in humans exposed to THS.

## Results and Discussion

We have developed a mouse model for THS exposure that approximates that of children and others in environments contaminated by THS (Materials and Methods). Materials commonly present in the homes and cars of smokers are exposed for specific periods of time to SHS from a smoking machine, 6 hrs/day, 5 days/wk for 24–26 wks, at a total particulate matter (TPM) of 30+/−5 µg/m^3^, a value that falls within the range detected by the Environmental Protection Agency (EPA) in the homes of smokers (15–35 µg/m^3^) [22], [23].

Direct comparison between biomarkers in our mice and in humans is difficult because population studies concerning THS-exposure in humans are just beginning. Moreover, these comparisons are difficult to make because humans do not always comply with the needed experimental constraints. Some of the components of SHS undergo chemical reaction with the indoor air to produce additional toxicants, at least some of which can be highly carcinogenic [1], [5], [7], [8], [24], [25]. Principal among these is nicotine that adsorbs on environmental surfaces and reacts with a ubiquitous environmental contaminant, nitrous acid, leaving those surfaces with potentially dangerous levels of NNA (1-(N-methyl-N-nitrosamino)-1-(3-pyridinyl)-4-butanal) and NNK (4-(methylnitrosamino)-1-(3-pyridyl)-1-butanone) [7], [8]. NNA is not found in SHS itself but NNK is. Furthermore, NNK is a lung-selective carcinogen and there has been considerable research on its toxicity and biomonitoring. A recent study *in vitro* showed that THS extracts and NNA itself are genotoxic to human cell lines [26].

To obtain a measure of the exposure to THS, we have measured NNAL (4-(Methylnitrosamino)-1-(3-pyridyl)-1-butanol), the principal metabolite of NNK. We chose to use nitrosamines to compare between humans and mice because the half-life of the nitrosamines (days) is much longer than that of cotinine (hours) and therefore makes the comparison between our well-controlled experiments and not-as-well-controlled human data collection more reliable [27], [28]. The intermittent exposure of children to cigarette smoke introduces uncertainty in cotinine data because the timing between the exposure and the urine collection for a study cannot be closely controlled. In contrast, nitrosamine data will average better over exposure time and urine collection time, producing a more reliable signal. We compared the levels in our mice with those we measured in a cohort of 50 SHS-exposed infants/toddlers aged 0.5 to 4 years [29]. The median NNAL level in the THS-exposed mice is 20% less than those of the SHS-exposed children (**Table 1**). The fact that we are exposing the materials to total particulate matter (TPM) levels similar to those detected by the EPA in the homes of smokers and the fact that we find the levels of a metabolite of NNK in the urine of the mice to be lower than those in children exposed to SHS (and inevitably also THS), gives us confidence that our THS exposure system is reliable and similar to that found in homes of smokers.

**Table 1:** Levels of NNAL (metabolite of NNK) in the urine of THS-exposed mice, humans exposed to SHS and in smokers.

Subjects	NNAL (pg.ml)
THS exposed mice, 6 months (our new data)	Range 16–84 Median = 35
Infants/toddlers, 0.5–4 years old (our new data)	Range = 1.4–283 Median = 44
SHS exposed humans	Range = 2–20
Smokers	Range = 400–600

**The range of NNAL levels in our THS-exposed mice fall within range we found in SHS- exposed infants/toddlers, with comparable medians.**

NNAL = 4 (Methylnitrosamino)-1-(3-pyridyl)-1butanol (*40).

At age 3 weeks, just-weaned wild-type mice (**not** genetically altered) are placed in standard, well-ventilated, mouse cages containing SHS-exposed materials. The animals are not constrained; they can move about the cage normally and the cages are kept in a large room with normal ventilation. Therefore, the environmental conditions also closely mimic those of THS exposure of children in the homes of smokers. These mice live in this environment for 6 months; materials exposed to THS are refreshed periodically as explained in the Materials and Methods. Control mice live in the same conditions but without THS exposure. At the end of 6 months, the animals are sacrificed and analyses performed.

**Figure 1:**
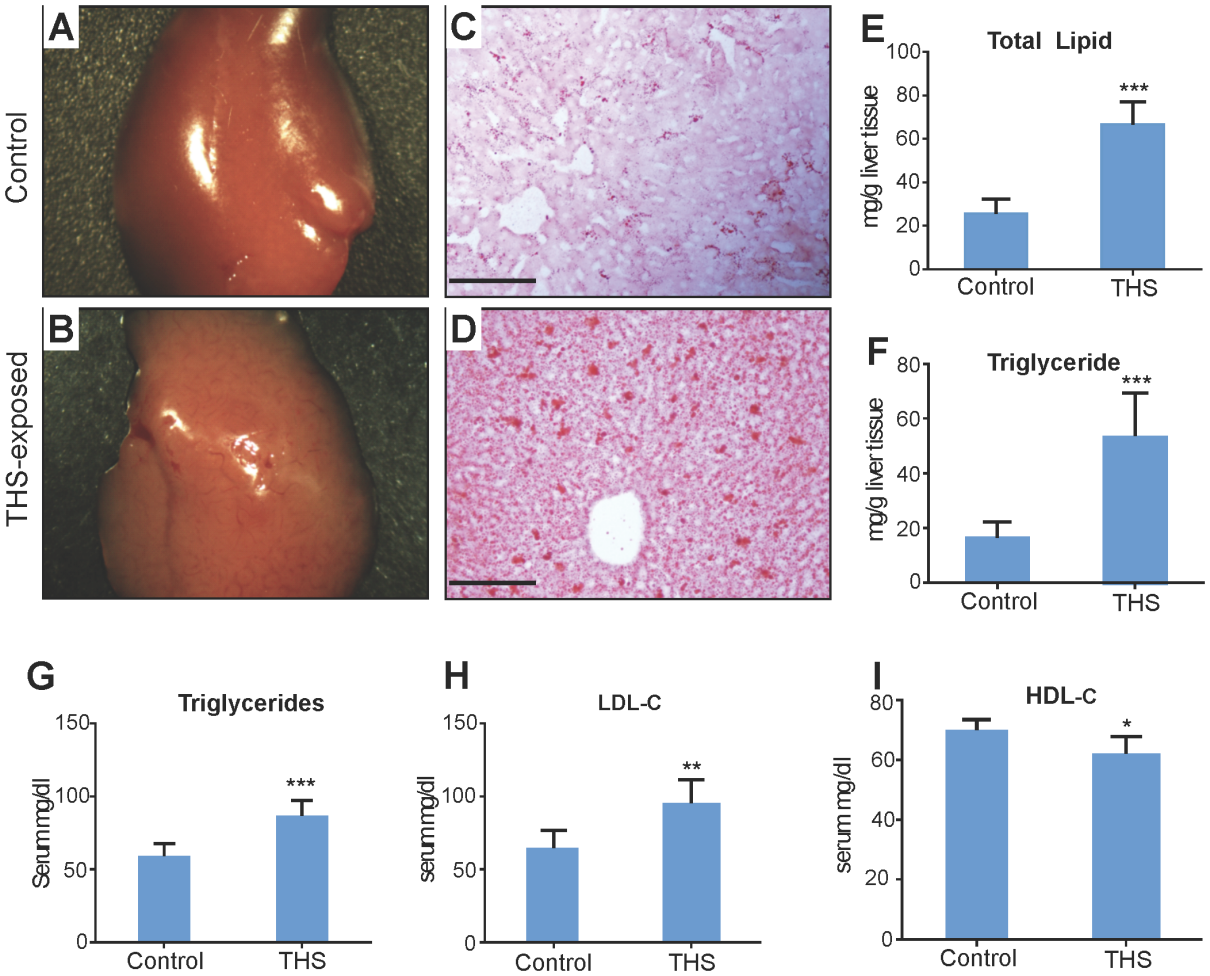
THS exposure results in non-alcohol fatty liver disease (NAFLD) with concomitant dyslipidemia. (**A,B**) Livers from mice exposed to THS for 24 weeks are a much paler pink than that of the control, an indication of fatty liver. (**C,D**) Liver cryosections from mice exposed to THS and stained with Oil-Red-O show increased lipid content and greater lipid aggregation than in the control. (**E**) Quantification of total hepatic lipid content shows lipid levels ∼2.5 times that of the control, with the majority of this increase being triglyceride accumulation (**F**). (**G**) Plasma triglyceride levels of mice exposed to THS were significantly increased. (**H,I**) Mice exposed to THS showed increased plasma LDL-Cholesterol and decreased HDL-Cholesterol levels. * p<0.05, ** p<0.01, *** p<0.001. Scale bars = 100 µm.

**In liver**, THS stimulates accumulation of fat in the hepatocytes (steatosis), giving the liver a pale red color compared to the deep red in normal liver (**Fig. 1A,B**). This occurs in 30% of the animals. Sections of liver tissue show small lipid droplets (red staining) in the control (**Fig. 1 C**) whereas in THS-exposed animals (**Fig. 1 D**), the ∼2.5-fold greater amount of lipid (**Fig. 1E**) coalesces into much larger droplets. There is also a greater increase of triglycerides (∼4-fold; **Fig. 1F**). Lipid elevation of more than 5% above normal fat indicates that steatosis has progressed to non-alcoholic fatty liver disease (NAFLD), a condition that, with prolonged exposure in humans, can lead to fibrosis, cirrhosis, and cancer. The blood of the animals exposed to THS shows significantly elevated levels of triglycerides and low-density lipoprotein (LDL, bad cholesterol) whereas high-density lipoprotein (HDL, good cholesterol) is significantly decreased (**Fig. 1 G–I**). These changes in liver metabolism have potential implications for cardiovascular disease and stroke [30]–[35].

The discovery that these mice have NAFLD, with abnormal hepatocyte function, suggests that they are susceptible to developing an impaired inflammatory response that could lead to fibrosis. This situation may aggravate drug-induced damage (e.g. by acetaminophen) at doses that normally would not be damaging. This is of particular concern for children because they are frequently treated with acetaminophen for fever and pain.

**Figure 2:**
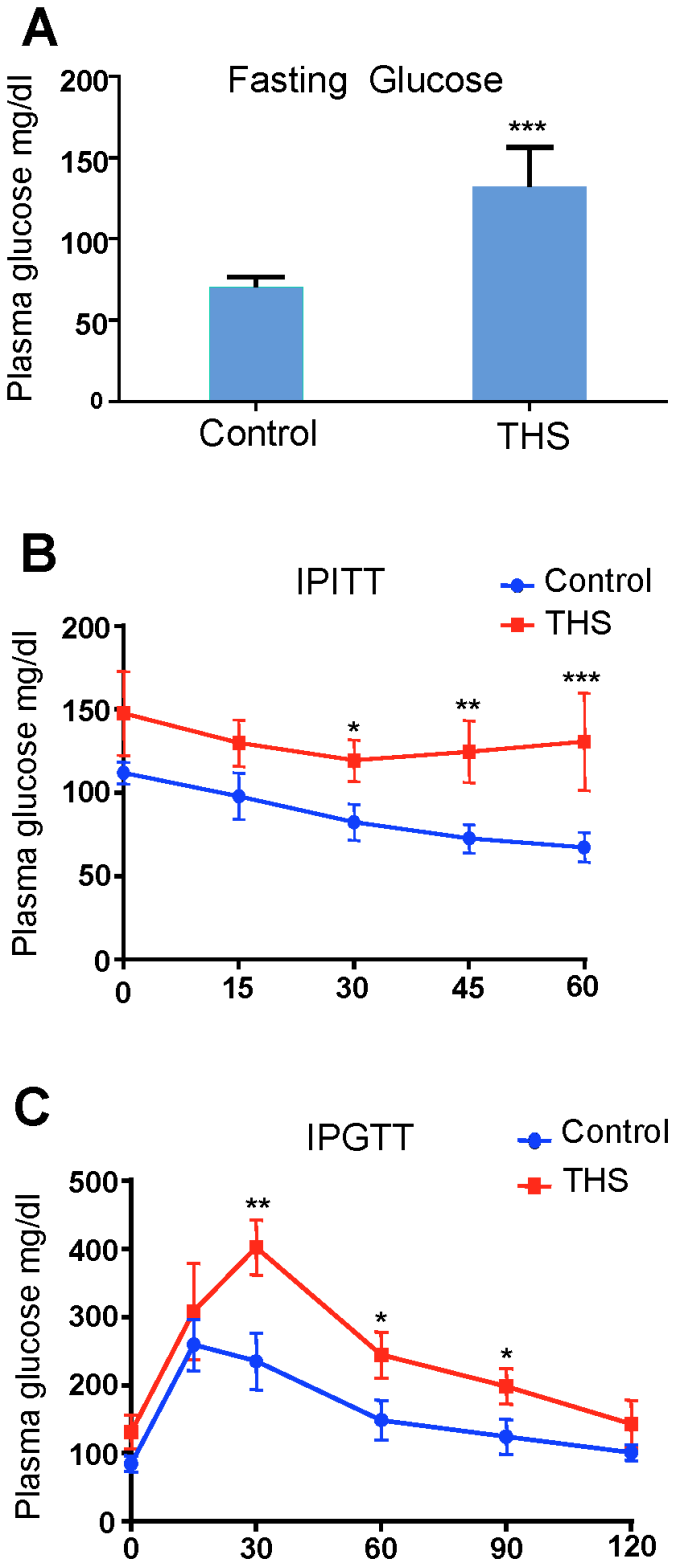
THS exposure results in hyperglycemia and decrease in insulin sensitivity. (**A**) Fasting glucose levels of mice exposed to THS were significantly increased in comparison to control. (**B**) Intraperitoneal Insulin Tolerance Test (IITT) time course and calculated area under the curve reveal that THS-exposed mice have decreased sensitivity to insulin which is highly correlated with both fatty liver disease and smoke exposure. (**C**) Intraperitoneal Glucose Tolerance Test (IGTT) time course shows impaired glucose clearance following glucose administration. *p<0.05, ** p<0.01, *** p<0.001.

A related important function of liver is regulation of insulin metabolism. We find that THS-exposed animals have fasting glucose levels that indicate they are pre-diabetic (**Fig. 2A**) and are significantly less efficient than control animals at using insulin to bring down blood glucose levels when an insulin tolerance test is performed (**Fig. 2B**). Moreover, a glucose tolerance test showed that THS-exposed mice handle the introduced glucose much less effectively than controls (**Fig. 2C**). The elevated triglycerides, increased LDL, decreased HDL and defects in insulin metabolism show that these animals are prone to developing “metabolic syndrome”, a condition that predisposes humans to stroke, coronary artery disease and type 2 diabetes [36]–[37]. These results are consistent with findings that show that tobacco smoke exposure and active smoking contribute to insulin resistance and could be associated with metabolic syndrome in US adolescent children [37].

**Figure 3:**
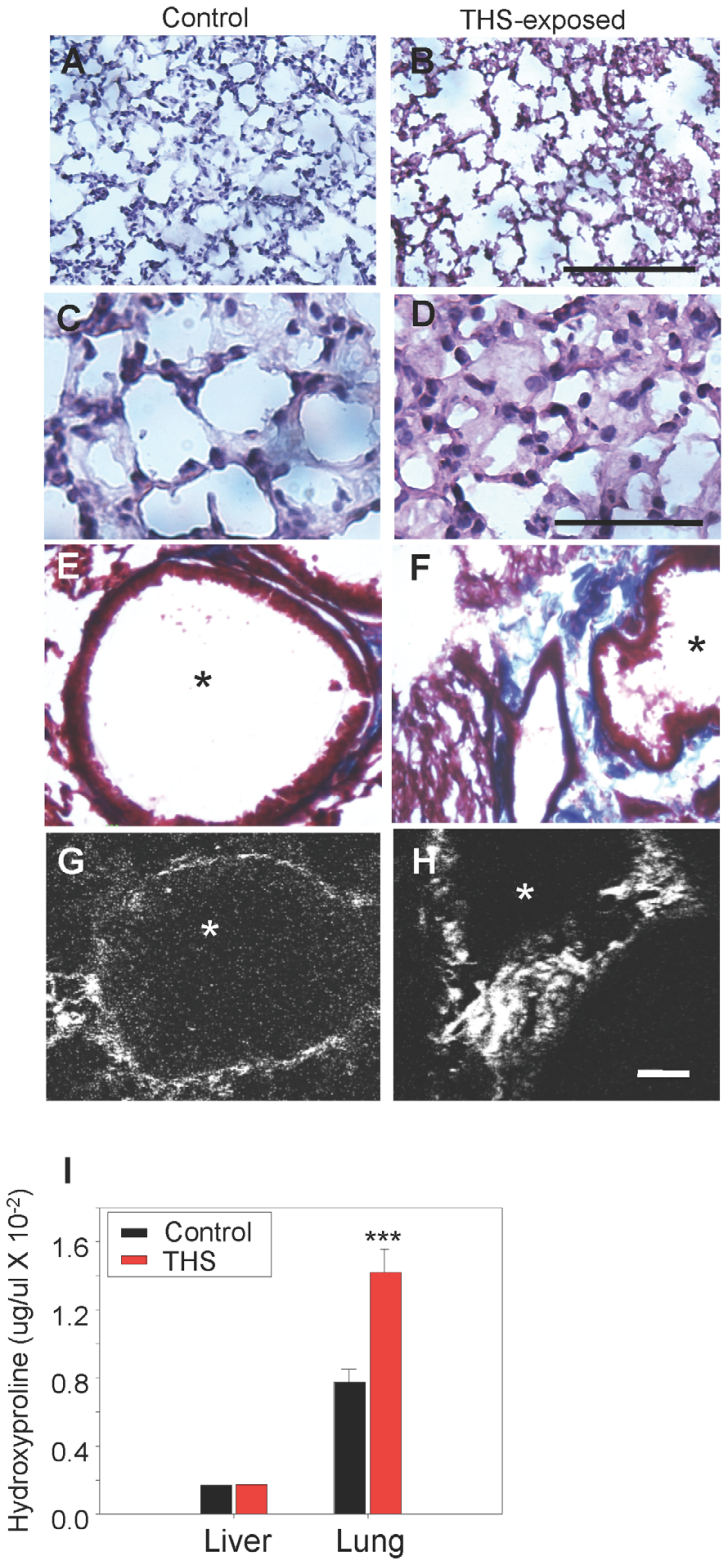
THS exposure results in excess deposition of collagen in lungs. Cross-sections through the lungs show that in THS-exposed animals, the alveoli in the region of the alveolar sacs are disrupted in comparison to the control animals (**A,B**). In the terminal respiratory bronchioles of the lung, however, the walls of the alveoli in the THS-exposed animals are thicker and appear to contain secretions (**C,D**). (**E–F**) Masson-trichrome staining for fibrillar collagen (blue) shows that the level of collagen in normal lung is low but THS-exposed animals show higher levels of fibrillar collagen with disrupted structure between alveoli (*). (**G,H**) Second-harmonic imaging microscopy (SHIM) confirms that collagen between alveoli (bright white) remains fibrillar in THS-exposed animals. (**I**) Hydroxyproline (an amino acid that is highly present in fibrillar collagen) is much higher in lung tissue of THS-exposed animals than in the control. Alveoli in E–H marked by *. Scale bar in **A,B** is 100 µm, in **C,D** is 50 µm, in **E–H** is 20 µm. In **A–H** and in **I**. *** p<0.001.

**Figure 4:**
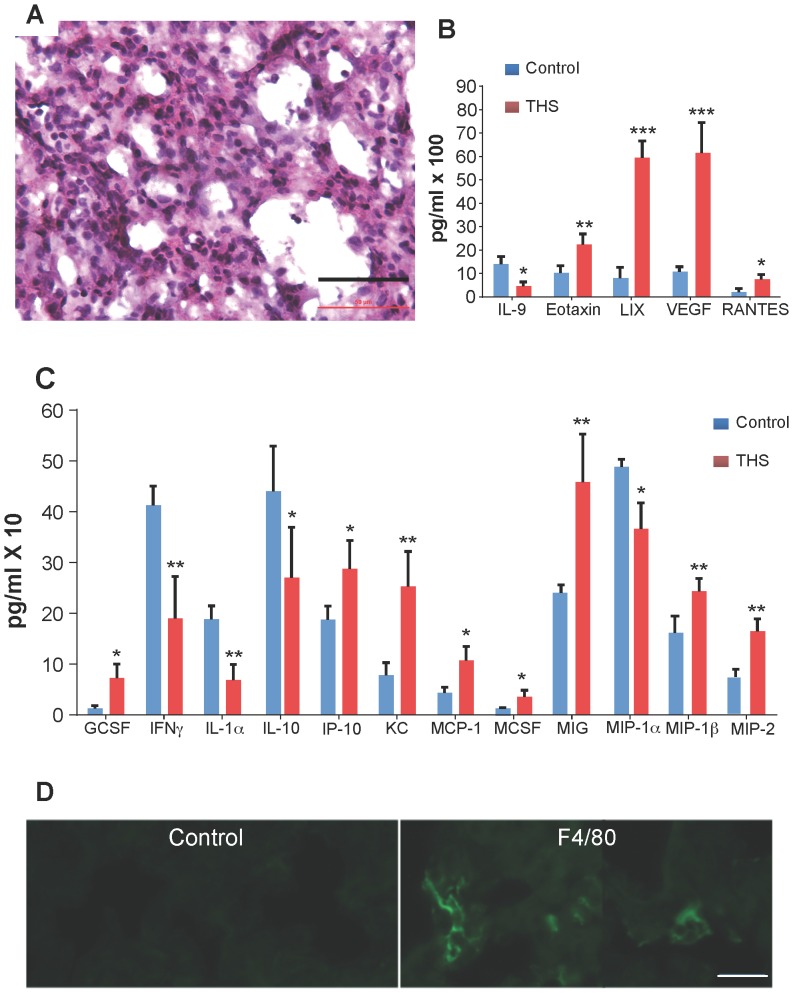
THS exposure results in inflammation and excess production of pro-inflammatory cytokines/chemokines in lung tissue. (**A**) Cross-section through the alveoli in the region of the terminal respiratory bronchioles shows that in THS-exposed animals there is significant inflammation in the tissue. (**B,C**) A multiplex cytokine array shows that many pro-inflammatory cytokines are elevated in THS-exposed animals (red) compared to control (blue) whereas some anti-inflammatory cytokines are decreased. (**D**) Lung tissue staining with an antibody for the F4/80 antigen that labels mouse macrophages. n = 3 for control; n = 5 for THS. Stars in B,C indicate * p<0.05, ** p<0.01, *** p<0.001. Scale bar in A = 50 µm and in D = 20 µm.

**In lung**, we observe that in the region of the alveolar sacs in THS-exposed mice the walls of the alveoli are disrupted significantly more often than in the controls (**Fig. 3A,B**) whereas the walls of the alveoli are thicker than those of the controls in the alveoli of the terminal respiratory bronchioles and some alveoli appear to contain secretions (**Fig. 3 C,D**). In the exposed mice, collagen fibers are disorganized but remain fibrillar (**Fig. 3E–H**) and the levels of collagen are higher (**Fig. 3I**). We also find that in some areas of the respiratory bronchioles the alveoli of the THS-exposed mice show cellular infiltration (**Fig. 4A**) whereas the controls do not (not shown). These observations suggest that the lung tissue could be producing pro-inflammatory cytokines. Indeed, we find that the pro-inflammatory cytokines/chemokines IP10, KC, MCP1, MCSF, MIG, MIP1β, MIP2, Eotaxin, LIX, VEGF are elevated whereas the anti-inflammatory cytokines/chemokines IL1α, IL9, IL10 are down-regulated (**Fig. 4B,C**), suggesting that there is a pro-inflammatory environment in the lungs. Indeed, we found significant numbers of macrophages and their presence was primarily in groups in the walls of the alveoli in the region of the respiratory bronchioles (**Fig. 4D**).

Our reproducible observation that the damage to the alveoli is different depending on the region of the lung is difficult to explain. We speculate that it could be due to the effects of oxidative stress induced by the THS toxins in regions with different cellular microenvironments. In the alveoli of the alveolar sacs, the oxidative stress may cause cell death whereas in the alveoli of the respiratory bronchioles it may stimulate fibroblasts to produce collagen. The elevated level of interstitial collagen, the thickened walls of some alveoli, the presence of macrophages in the walls of those alveoli and the increase in pro-inflammatory cytokines suggest an increased risk for development of fibrosis in people who have been exposed to THS for prolonged periods of time. Fibrosis in lung ensues when fibrotic tissue replaces the parenchymal tissue. The consequence is scar tissue formation and decreased oxygen diffusion rate. It is possible that THS-exposed people have a lower tolerance for drugs that induce lung fibrosis, given that they already have a propensity for development of this condition [38]–[40]. Doctors should take this into consideration when designing treatments for individuals who have been living in environments in which THS is a contaminant (e.g. spouses and, potentially, elderly parents of smokers).

**Figure 5:**
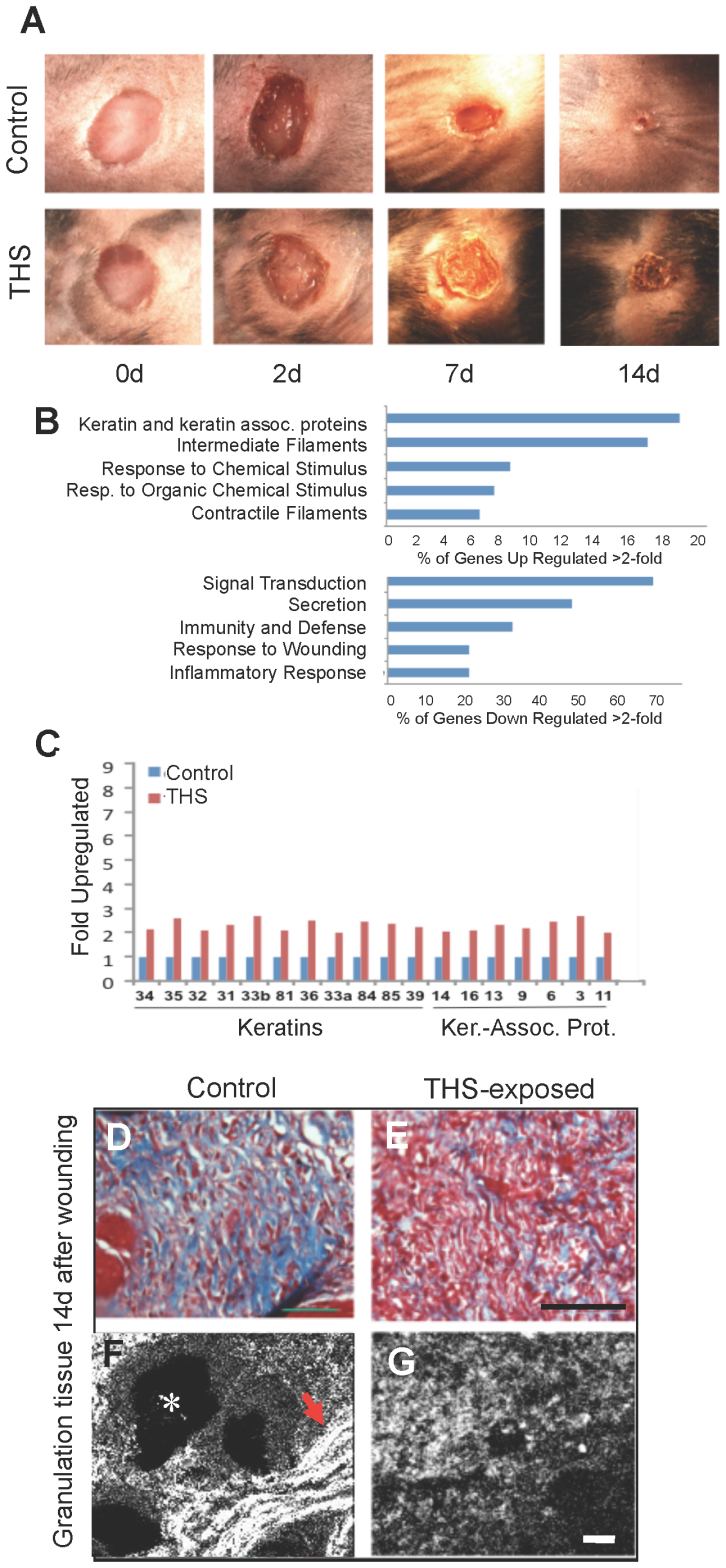
THS exposure delays closure of and weakens cutaneous wounds. (**A**) Representative excision wounds performed on the backs of mice after hair removal show that THS exposure results in keratinization of the epithelium (crusty appearance) and delayed wound closure. (**B**) At day 14, when control wounds are closed, gene expression evaluated by Affirmetrix Arrays shows that THS exposure upregulates keratin genes and genes involved in epithelial migration and contractile function of wound tissue and downregulates genes involved in inflammatory and immune responses. (**C**) The upregulated keratin genes are primarily associated with hair and nail production, resulting in stiffening of the wound, making it crusty and potentially more fragile. “Ker.-Assoc. Prot.” = Keratin-Associated Protein. (**D–G**) In cross sections through the healing tissue, Masson-trichrome staining shows great decrease of interstitial collagen (blue) in THS-exposed tissue; second-harmonic imaging microscopy (SHIM) shows that the collagen is strongly fibrillar in the control (arrow) but not at all fibrillar in THS-exposed mice. For **A**; for **B,C**, for **D–G**. Scale bar for **D,E** = 100 µm and for **F,G** = 20 µm. White * labels a hair follicle.

**In skin**, wounds take longer to heal in THS-exposed mice (**Fig. 5A**) and show characteristics that are conducive to reopening, such as heavy keratinization of the epithelium. The expression of numerous genes for keratins and keratin-associated proteins that are normally produced for hair and nails is increased (**Fig. 5B** **, upper panel, C**). Moreover, the expression of genes that are important in the inflammatory response and response to wounding is decreased (**Fig. 5B** **, lower panel**). In the healing tissue, the level of fibrillar collagen (blue) is greatly decreased in THS-exposed animals (**Fig. 5D,E**). The majority of collagen is not fibrillar and appears to be degraded (**Fig. 5F,G**), an observation consistent with gene array analysis showing a decrease in expression of tissue-inhibitor metalloproteinase 1 (TIMP1), an inhibitor of matrix metalloproteinases. This is in contrast to the lung where the fibrils of collagen are still intact, albeit disorganized. These observations could explain why, in the case of the lung, fibrosis develops whereas, in the skin, the lack of fibrillar collagen could lead to poor healing. These differences would be due to the fact that the lung suffers a chemical injury and the skin a chemical and mechanical injury.

It has long been known that smokers' wounds heal poorly [41]. This is particularly important when they undergo surgery. As a consequence, surgeons commonly recommend or require cessation of smoking for at least four weeks prior to surgery. Evidence shows that the early effects of smoking on blood-vessel constriction are reversible in less than an hour after smoking whereas the deficiencies in the inflammatory response do not return to normal until ∼4 weeks after cessation [41] and it is not known how long it takes for the damage to cells to be reversed. Indeed, many times surgical wounds reopen even if the patient stopped smoking well before surgery. It is not yet clear why this occurs. Our work shows that not only smokers but also those exposed to SHS and THS may suffer from these wound-healing risks. The delay in wound closure accompanied by the presence of decreased fibrillar collagen (**Fig. 5D–G**) in the healing tissue results in marked reduction of strength of wound tissue. This, in conjunction with the presence of keratins that convey rigidity to the epithelium and cells rich in contractile filaments, could be the cause for reopening of surgical wounds in smokers and, potentially, for those exposed to SHS and THS.

**Figure 6:**
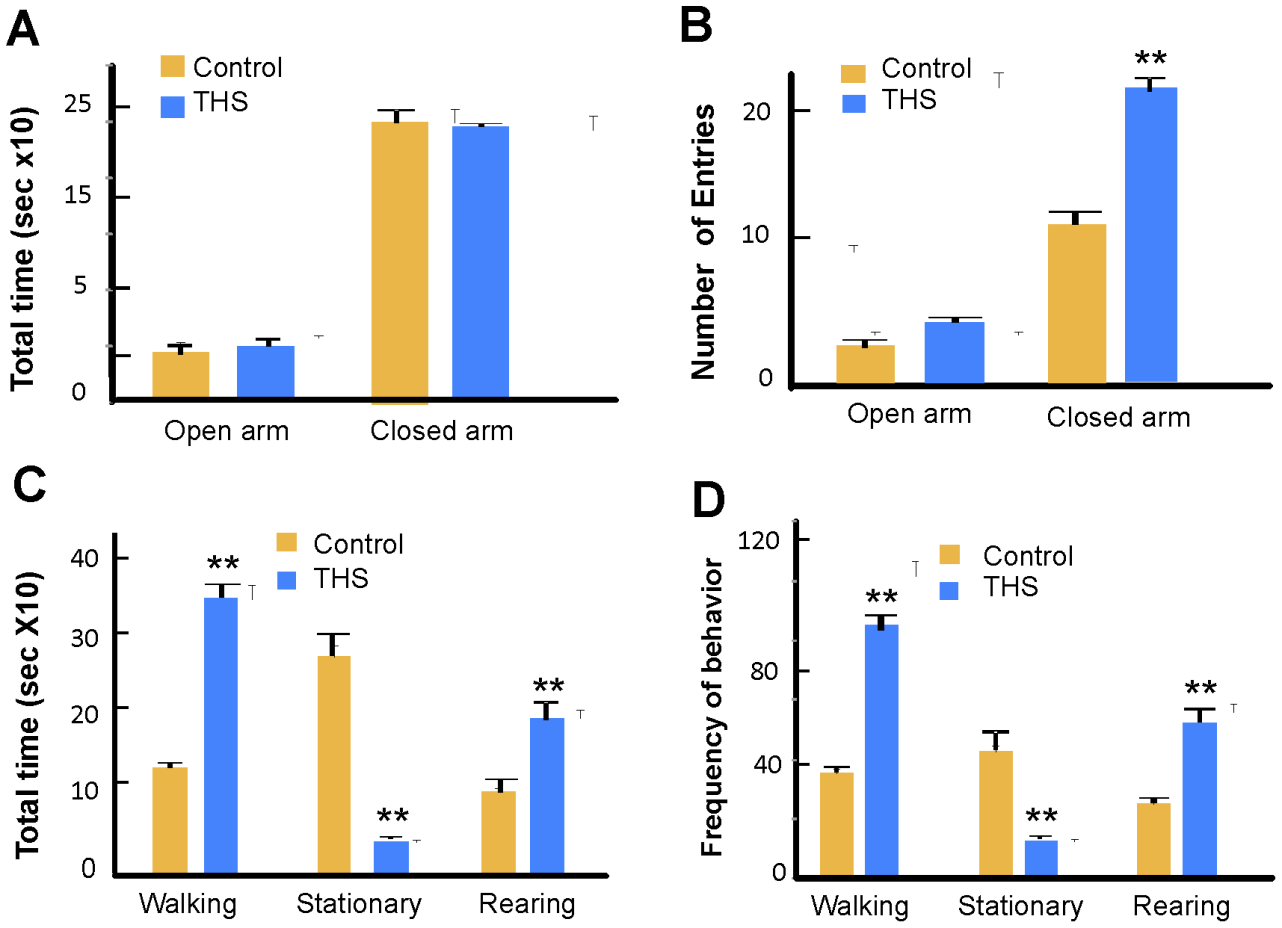
Effects of THS-exposure on behavior of mice. (**A,B**) *Testing for anxiety*. Control and THS mice were tested using an Elevated Plus T-maze (**Fig. S1A**). (**A**) Total time spent in the open and closed arms was measured. The two groups both spent much more time in closed than in open arms of the maze, indicating normal anxious behavior. (**B**) The frequency of entries into the closed arms was significantly greater for THS-exposed as compared to control mice, indicating more locomotor activity. n = 6 controls and 6 THS-exposed mice. (**C,D**) *Testing for hyperactivity*. The Open Field test (**Fig. S1B**) was used to perform these experiments. The behavior of control and THS-exposed mice was observed for 1 hr. (**C**) The THS mice spent much more time walking, much less time stationary, and more time rearing than the controls. (**D**) The same general pattern was observed for the frequency of transition between these behaviors; n = 12 controls and 12 THS-exposed mice.

**In behavior**, THS-exposed animals showed behavior that seemed anxious or hyperactive. Therefore, we performed a standard test that is designed to examine anxiety [42] – the “Elevated Plus” T-maze (**Fig. S1A**). Time spent in the closed arms represents anxious behavior; rodents generally spend much more time in the closed arms. THS-exposed mice and controls display the same level of anxious behavior, as illustrated by their similar preference for closed over open arms of the maze (**Fig. 6A**). In contrast, when the frequency of entry into the open and closed arms was scored, the THS-exposed mice showed a significantly higher frequency of entries into the closed arms than did the control (**Fig. 6B**), suggesting that THS-exposed mice may be hyperactive. To test this possibility, we used the Open Field test (**Fig. S1B**). Individual mice were placed in the Open Field; walking, stationary and rearing behaviors were assessed, as well as the frequency of transition from one of these behaviors to another. THS-exposed mice spent significantly more time walking, much less time standing still and more time rearing than control mice (**Fig. 6C**). The frequency of transitions between these behaviors shows a similar pattern (**Fig. 6D**). In particular, THS-exposed mice were almost constantly in motion whereas control mice were stationary for a considerable fraction of the time. We illustrate this comparison with two movies, one of a control mouse (**Video S1**) and the other of a THS-exposed mouse during the first 10 minutes of the videotaped hour (**Video S2**). These movies are sped up by a factor of two to better illustrate the differences in activity during this period (the movies are found in the Video S1 & S2).

**Figure 7:**
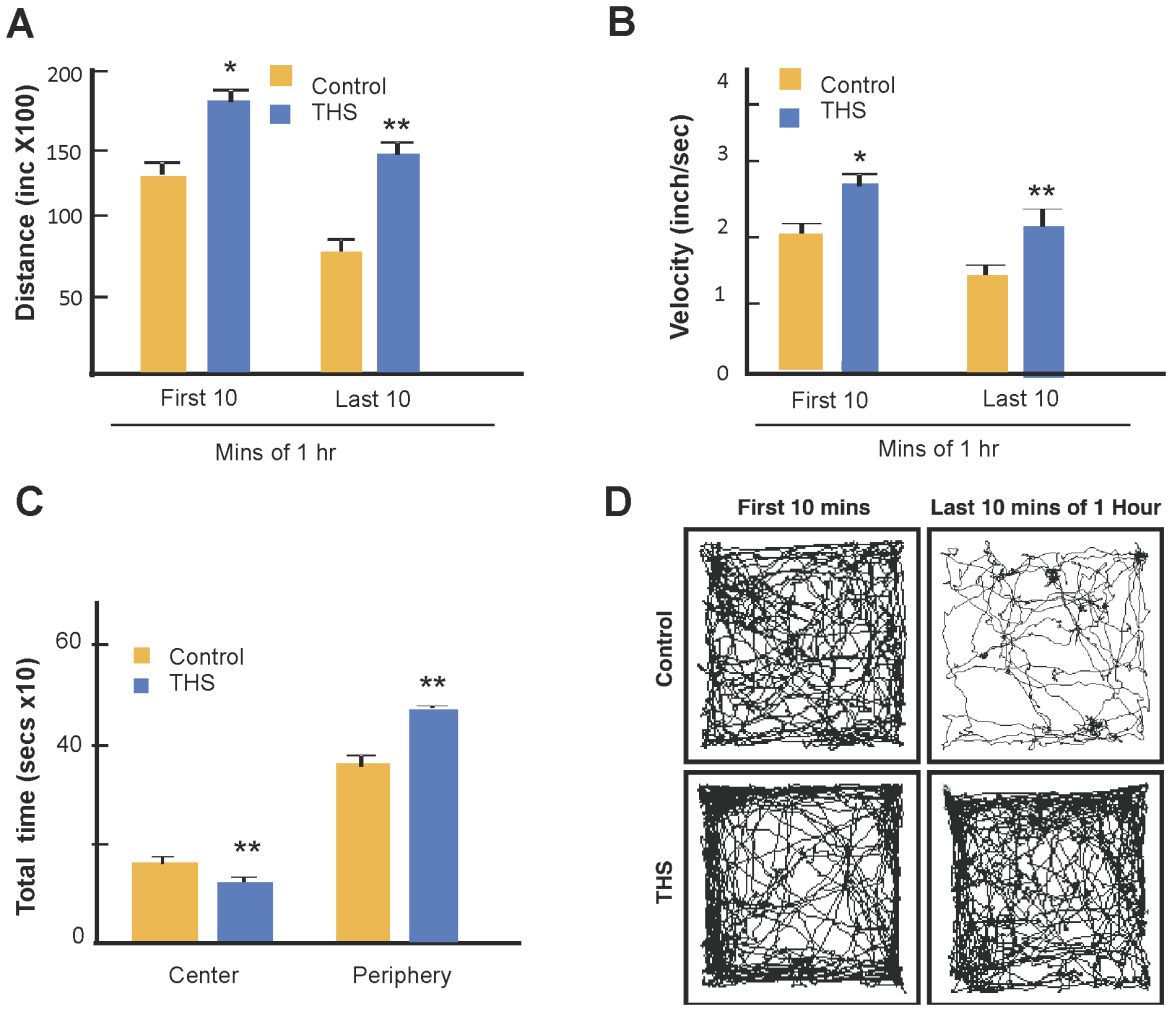
Effects of THS-exposure on hyperactive behavior. (**A**) Distance and (**B**) velocity of travel during the first and last 10 minutes of the 1 hr test. (**C**) Time spent in center of the field vs. the periphery during the first 10 minutes. (**D**) Raster plots showing the integrated paths that a control mouse (top) and a THS-exposed mouse (bottom) traveled in the first 10 mins and last 10 mins of the hour. The plots were chosen based on the average distance traveled by each cohort of control and THS exposed mice. The Ethnovision software produced the raster images for each individual mouse along with an Excel sheet containing the distance traveled by each mouse as represented by the raster image. The mean distance traveled for each cohort was then calculated and, based on that mean, the raster image representing the path traveled by the single mouse closest to the cohort mean distance traveled is shown. See also video s1 showing the behavior of the control and video s2 showing the behavior of the THS-exposed mice. n = 12 control and 12 THS-exposed mice. * p<0.05, ** p<0.01.

For confirmation, a second set of mice was tested in the Open Field and using Ethovision 7.1 video tracking software, we tracked mice individually for an hour. Again, it was seen that the THS-exposed mice covered longer distances (**Fig. 7A**) at higher velocities (**Fig. 7B**) and spent significantly more time in the periphery of the field (**Fig. 7C**). The difference in behavior between the two groups was particularly striking in the first two minutes during which the THS-exposed mice moved on average at high but decreasing velocity (**Fig. S2**) and the last 10 minutes of the hour in which the control mice showed on average little activity whereas the THS-exposed mice remained very active (**Fig. 7D**). We conclude that THS-exposed mice are hyperactive.

These data are consistent with previous findings in humans that link hyperactivity to tobacco smoke exposure [43], [44]. Examination of the 2007 National Survey on Children's Health of 55,358 children under the age of 12, found that 5.6% had attention-deficit/hyperactivity disorder (ADHD), 8.6% had learning disabilities and 3.6% had behavior and other conduct disorders but children who were exposed to SHS at home (and therefore unavoidably also exposed to THS) had a 50% greater chance of having 2 or more neurobehavioral disorders than children not exposed. It was also found that children 9–11 living in poverty were at even higher risk of tobacco-smoke associated neurobehavioral disorders [45], [46]. Even very low-level exposure is associated with cognitive deficits in children [47].

### Conclusions

The most important finding of this study is the clear similarity between our results in THS-exposed mice under conditions that simulate human exposure and the studies in humans described above. This is particularly evident in the hyperactive behavior of THS-exposed mice that validates previously existing correlative data in children exposed to SHS/THS and the impaired healing in THS-exposed mice that parallels the reluctance of surgeons to operate on smokers for fear of their surgical wounds reopening [41]. Also very important is that THS-exposed mice exhibit changes in liver metabolism that, in humans, have important implications for development of metabolic syndrome, a condition that predisposes humans to stroke, coronary artery disease and type 2 diabetes [34]–[36]. In the lung, the combined alterations in the alveoli and the elevation of pro-inflammatory cytokines suggest an increased risk for fibrosis, with potential consequences for tissue scarring and decreased oxygen diffusion. Lastly, in terms of real world consequences for children of smoking parents, a recent study showed that children living with 1–2 adults who smoke in the home, where SHS and its residues (THS) are abundant, were absent 40% more days from school due to illness than children who did not live with smokers [48].

There is still much to learn about the specific mechanisms by which cigarette smoke residues (THS) harm nonsmokers but that there is such an effect is now clear. Our studies in mice that are never exposed to smoke itself but are exposed to residues of the smoke, strongly implicate tobacco smoke residues in these pathologies. It follows that children in environments where smoking is, or has been allowed, are at significant risk for suffering from multiple short-term and longer health problems, many of which may not manifest fully until later in life.

## Materials and Methods

### Animals

C57BL/6 mice were divided into control and experimental groups. The experimental group was exposed to THS from right after weaning to 24 weeks; the control group was never exposed to THS. All mice were fed a standard chow diet (percent calories: 58% carbohydrate, 28.5% protein, and 13.5% fat).

### Ethics Statement

Animal experimental protocols were approved by the University of California, Riverside, Institutional Animal Care and Use Committee (IACUC). The animal use protocol is A-2008024.

### THS Exposure

Using the Teague smoking apparatus [49], common household fabrics were placed in mouse cages and subjected to SHS. Each cage contained 10 g of curtain material (cotton) 10 g of upholstery (cotton and fiber) and two 16in^2^ pieces of carpet (fiber) to maintain equal exposure levels across experimental groups. Two packs of 3R4F research cigarettes were smoked each day, 5days/week and smoke was routed to a mixing compartment and distributed between two exposure chambers, each containing 8 cages with the materials. We use the gravimetric method to determine the particulate concentrations. Whatman grade 40 quantitative cellulose filter papers are first weighed, then introduced into the filtering device and, after running the test for 15 mins, the filter is weighed again to determine the particulate mass that has accumulated during this time. This procedure is repeated with 2 more filters and the average of the 3 masses calculated gives the TPM values for each chamber. All cigarettes were smoked and stored in accordance with the Federal Trade Commission (FTC) smoking regimen [50]. At the end of each week, cages were removed from the exposure chamber, bagged, and transported to the vivarium where mice were placed into the cages. For the next week, an identical set of cages and fabric was then prepared and exposed to smoke in the same way as described above. By using two sets of cages and material, each of which were exposed on alternating weeks, we ensured that mice inhabited cages containing fabric that had been exposed (according to the regimen described above) to fresh SHS during the previous week. Throughout the exposure period, hair was removed weekly from the backs of the mice to mimic the bare skin of humans.

Control animals were tested for behavior and wound healing in a pilot study to look for possible changes related to living in the (non-exposed) materials of this project. No significant differences were observed in relation to animals living in normal conditions in the vivarium. During these experiments, in all cases the cages for THS-exposed and control mice were the same, food was the same, temperature the same, etc. Hair was not removed in this particular set of experiments but we have removed the hair in control mice in many other studies of dermal exposure to tobacco chemicals and found no differences between controls with and without removed hair.

### Histology

Tissues were fixed in 4% paraformaldehyde overnight. Following fixation, tissues were rinsed in PBS, incubated in 0.1 M glycine solution, cryoprotected by two successive incubations with 15% and 30% sucrose solution and embedded in optimal cutting temperature (O.C.T.) embedding medium. Liver sections were stained for 15 min in freshly prepared Oil-Red-O solution (0.3% Oil-Red-O in 3∶2 solution of isopropanol and H_2_O) to visualize lipid or stained with Diff-Quick to visualize the structure of the tissue. Lung and skin sections were stained with Diff-quick for structure, Masson-trichrome for fibrillar collagen and examined by second harmonic generation imaging (SHIM) to evaluate the structure of the fibrillar collagen.

### Liver Lipid Analysis

To quantify the total lipid content of the liver, the Folch method [51] was used. Livers were homogenized with a 2∶1 solution of chloroform and methanol. Following centrifugation, the aqueous phase was washed with 1.0% NaCl solution, centrifuged, and the upper aqueous phase removed. The remaining lower phase containing the lipid was dried under a nitrogen stream and the lipid content weighed to determine total hepatic lipid level. The triglyceride content was determined using a kit developed by AbCam (Cat #ab65336). Liver tissue from THS-exposed mice was homogenized in 1% Triton X-100 and pure chloroform solution, centrifuged, and the lower organic phase collected, dried, and mixed in assay buffer for colorimetric assay (O.D. 570 nm).

### Plasma Lipid Analysis

Following a 6 hour fast, blood was taken via cardiac puncture and allowed to coagulate. Samples were centrifuged at 6,000 rpm and plasma collected. To quantify HDL-cholesterol, LDL-cholesterol, and triglyceride concentrations, commercially available kits developed by Abcam (Cat #ab65341 & #ab65336) were utilized. Plasma samples were prepared according to the manufacturers guidelines and measured at 570 nm.

### Blood Glucose Analysis

Fasting blood glucose (FBG) levels were measured using a commercially available kit (TRUEresult) and gold sensor-laser accuracy strips. Mice were fasted for 12 hrs for obtaining FBG levels. A nick was made on the tail vein and the FBG level was measured using the strip brought into contact with a drop of blood from the nicked tail vein. Intraperitoneal insulin tolerance test (IPITT) was performed on mice fasted for 12 hrs. Blood glucose reading was taken at time 0 followed by humulin (recombinant human insulin) injection at a dose of 10 ml/kg body weight. Humulin solution was prepared from stock humulin at a 1∶2000 dilution in saline. Glucose levels were taken every 15 min for the following 60 min. Intraperitoneal glucose tolerance test (IPGTT) was done following a 12 hr fast. Blood glucose was taken at time 0 followed by glucose administration. Mice received 20% glucose solution at a dose of 10 ml/kg body weight.

### Luminex multiplex array assays

Lung tissue was homogenized in RIPA at 4°C for 5–10 minutes. Protein concentration in the tissue was determined, the assays conducted according to the manufacturers' protocols (Invitrogen, Carlsbad, CA, and Millipore, Billerica, MA) and read using a Luminex™ 100 instrument (Invitrogen, Carlsbad, CA), which quantifies cytokine levels by monitoring R-Phycoerythrin RPE fluorescence associated with bead sets.

### Second Harmonic Generation (SHG) imaging

An inverted Zeiss LSM 510 non-linear optics (NLO) META laser scanning microscope equipped with standard illumination for transmitted light and epi-fluorescence detection was used. The microscope was also equipped with an NLO interface for a femtosecond Titanium:Sapphire laser excitation source (Chameleon-Ultra, Coherent, Incorporated, Santa Clara, California) for multi-photon excitation. The Chameleon laser provided femtosecond pulses at a repetition rate of about 80 MHz, with the center frequency tunable from 690 to 1040 nm. A long working distance objective (Zeiss, 40× water, N.A. 0.8) was used to acquire the images. The two-photon signals from the sample were epicollected and discriminated by the short pass 650 nm dichroic beamsplitter. The SHG images were collected using a META detection module with signals sampled in a 394–405 nm detection range (λ_ex_ = 800 nm). Each image presented in this work is 12 bit, 512×512 pixels representing 225 µm×225 µm field of view.

### Hydroxyproline assay to measure interstitial collagen levels

Tissue hydroxyproline was measured by thiobarbituric acid reactive substances using a commercially available kit (BioVision Inc., Milpitas, USA). Tissue samples were homogenized in water and hydrolyzed with HCl in pressure-tight Teflon capped vials at 120°C for 3 hrs. Hydrolyzed samples were dried in a 96 well plate in a hot air oven at 70°C followed by addition of chloramine T and dimethylaminoborane and incubated for 90 mins at 60°C. The hydroxyproline forms a chromogen with an absorbance maximum at 560 nm.

### Mouse wounding experiments

Experiments were approved by the Institutional Animal Care and Use Committee of the University of California, Riverside. After removal of dorsal hair, excision wounds were created on the dorsum of both control and THS-exposed mice using a 7 mm biopsy punch (Acuderm, Inc). Wound tissues were collected using a 10 mm diameter Acupunch at various time points following injury. Half of each sample was homogenized for the Affimetrix array assays and the remainder prepared for frozen sections.

### Affymetrix microarray and data analysis

We used Affymetrix Mouse Genome 430 2.0 Arrays, which covers over 39,000 transcripts on a single array. Wound tissues were collected at 14 days post-healing and total RNA extracted and evaluated for quality using the Agilent Bioanalyzer 2100 using the Agilent RNA 6000 Nano Assay kit (Agilent Technologies, Waldbronn, Germany) and the concentration determined using the NanoDrop ND-1000 spectrophotometer (NanoDrop Technologies, Inc., Wilmington, DE USA). After the arrays were performed, a single log_2_ expression measure for each probe set was calculated from image files (CEL format) using the robust multi-array analysis (RMA) procedure using Agilent GeneSpring GX software. The changes of expression level between non-exposed and exposed wound tissues were compared. Only genes that were over- or under-expressed by >2-fold were considered.

### Elevated Plus Maze

Singly-housed mice were transferred from the vivarium to the test room the morning of the trials. The elevated plus maze was manufactured in house as described previously [42]. Briefly, arms were constructed out of black acrylic plastic measuring 3.5 inches wide by 44 inches long and stood 3 feet off the floor (**Fig. S1A**). The two arms that were closed had 8.5 inch black plastic walls. The maze was used in a quiet room and was enclosed by a black curtain with low light. All trials were video recorded. At the beginning of each trial, mice were placed in the middle of the maze facing a closed arm. The duration of time spent in the open or closed arms and the frequency of entries into open and closed arms were scored using the event recorder JWatcher (freeware Blumstein's Laboratory, UCLA).

### Open Field Test

To test for hyperactivity we used the Open Field test [52]. The apparatus consists of a chamber made from plexiglass and covered with white vinyl to increase the contrast to better visualize the dark mice (**Fig. S1B**). Six control and six THS mice were used. Each mouse was placed in the center of the apparatus and videotaped for one hour. Analysis was performed during the first 10 min using the event–recorder JWatcher program. Rearing, Stationary and Walking behaviors were assessed, as well as the transition from one of these behaviors to the next and frequency of transition. A mouse was considered hyperactive if it spent more time walking than stationary and had more frequency of behavior transitions from one behavior to the next. To confirm these findings, the experiment was repeated; another set of 6 control mice and 6 THS-exposed mice were tested for 1 hr. This time the analysis was performed using Ethovision 7.1 video tracking software and the first 10 min and last 10 min of the tape were analyzed for distance and velocity of movement of each mouse. The software also produced raster plots that showed tracings of the movements of the mice superimposed on the image of the maze. The tracings shown in Fig. 7 are for a representative mouse for each cohort. The software also enabled us to define the center and periphery of the maze as different zones and then the software calculated the distance traveled in each respective zone [52].

### Determination of 4-(methylnitrosamino)-1-(3-pyridyl)-1-butanol (NNAL) in Urine

Determination of the NNK metabolite NNAL was carried out using liquid chromatography – tandem mass spectrometry (LM-MS/MS) [27].

### Statistical Analysis

Significance was determined using Student's t-test for comparison between 2 means and ANOVA for comparison between more than 2 means. All data were examined to assure homogeneity of variance. Means were considered significantly different when p<0.05.

## Supporting Information

Figure S1 **Schematic representation of the apparatus used to compare the behavior of normal vs THS-exposed mice.** (A) Elevated Plus maze, used to assess anxiety. (B) Open Field Test Used to test levels of hyperactivity.
(TIF)

Figure S2 **Quantification of data in** **Fig. 7D** **: Graphs of the distances traveled (A) and (B) velocities of the mice whose tracks are shown in** **Fig. 7D** **.** Yellow is the THS exposed mouse and the blue is the control normal mouse. Note that the THS-exposed mouse was running at a great speed initially but quickly slowed down to a velocity only slightly greater than the control. However, with time, the control slowed down significantly whereas the THS-exposed mouse remained approximately constant in activity.
(TIF)

Video S1 **Control mouse 1 (2× speed)-Control non-exposed mouse in the open field chamber to test for hyperactivity.** The speed of this video has been increased by two times for faster viewing purposes. The control mouse moves more slowly and less frantically than the THS-exposed mouse. It also has longer rest periods and stays and travels more in the center of the chamber, as is expected of a mouse that is not hyperactive. This mouse is representative from an n = 12.
(ZIP)

Video S2 **THS mouse#1 (2× speed)THS-exposed mouse in the open field chamber to test for hyperactivity.** The speed of this video has been increased by two times for faster viewing purposes. The THS-exposed mouse moves faster, with fewer rest periods and appears to be frantic. Furthermore, the THS-exposed mouse stays along the periphery of the chamber and rarely travels or goes through the center of the chamber, a characteristic found in hyperactive behavior. This mouse also puts its front paws up throughout the test as if it is trying to find a way to escape out of the chamber. This mouse is representative from an n = 12.
(ZIP)
