# Expression of Concern: Cigarette Smoke Toxins Deposited on Surfaces: Implications for Human Health

**DOI:** 10.1371/journal.pone.0289796

**Published:** 2023-08-03

**Authors:** The PLOS ONE Editors

A representative of UC Riverside has contacted the *PLOS ONE* Editors to notify us of concerns related to this article [1] following an institutional investigation into concerns raised about laboratory practices and recordkeeping related to the operation and maintenance of the smoking machine used in this study.

The *PLOS ONE* Editors have followed up with the corresponding author, who stated that total particulate matter (TPM) measurements were accurately recorded during the study period and repairs made to the smoking machine were documented; however, records from the period in question are no longer available. The corresponding author stated that they had access to and reviewed the raw data prior to submission of the article, but indicated that not all data remain available. The last author has indicated that while TPM records are no longer available, records of the maintenance and repairs of the smoking machine, as well as the operation protocol used in the lab, are available, and that the underlying data for the behavioral experiments (Figs 6 and 7) and the Affymetrix array data remain available. The editors were unable to clarify the extent of the unavailability of the underlying data at the time of preparation of this notice.

The *PLOS ONE* Editors issue this Expression of Concern to inform readers about the concerns that have been raised but could not be fully resolved in editorial follow up.

PLOS has been informed that litigation was initiated by the corresponding author against UC Riverside and at the time of this notice’s publication the litigation remains pending.
