# Supplementary figures and images for: Cigarette Smoke Toxins Deposited on Surfaces: Implications for Human Health
Source: PLoS One. 2014 Jan 29;9(1):e86391. doi: 10.1371/journal.pone.0086391 (PMC3906039; doi:10.1371/journal.pone.0086391)

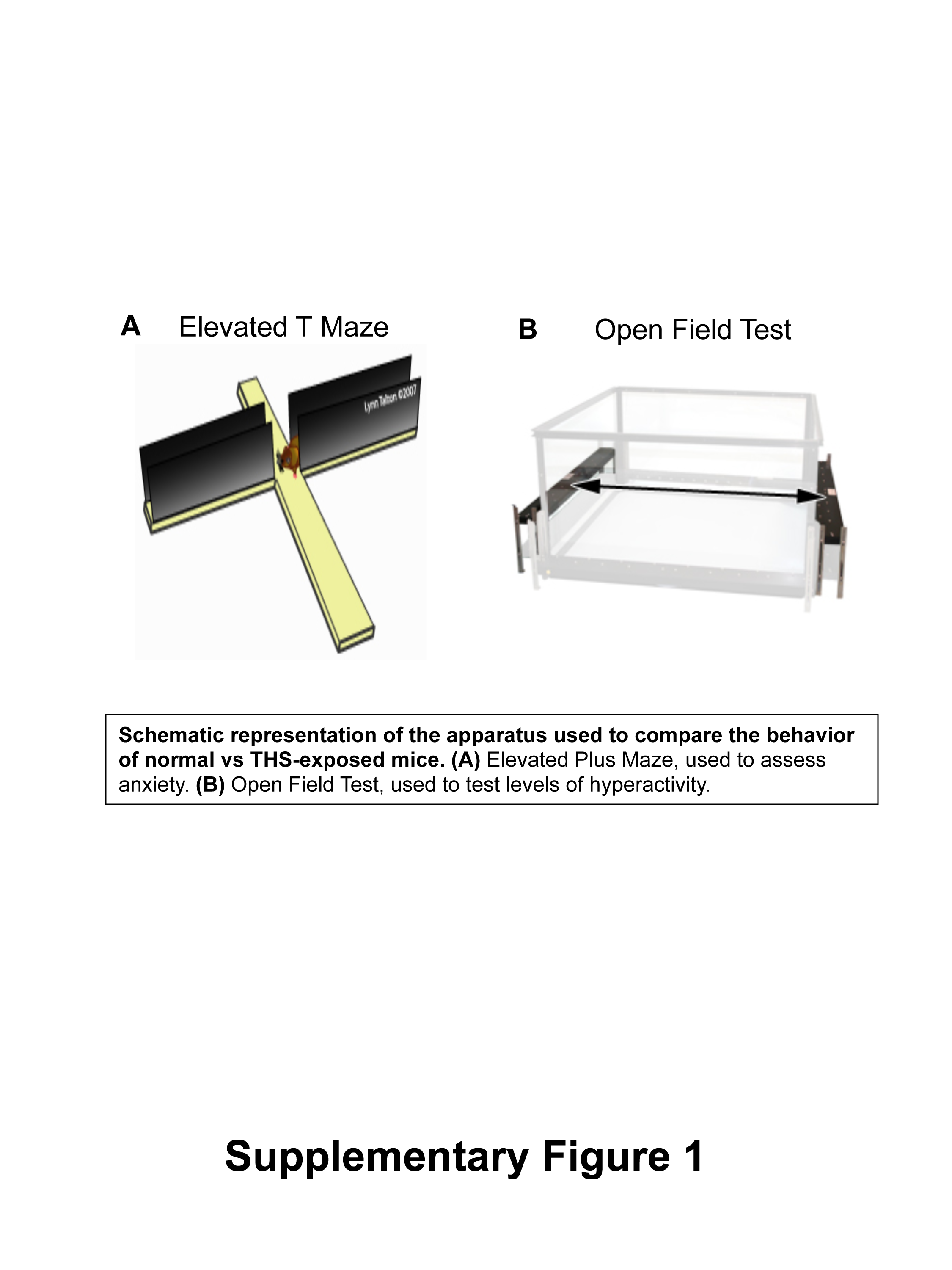

Supplement: Figure S1 — Schematic representation of the apparatus used to compare the behavior of normal vs THS-exposed mice. (A) Elevated Plus maze, used to assess anxiety. (B) Open Field Test Used to test levels of hyperactivity. (TIF) [file pone.0086391.s001.tif]

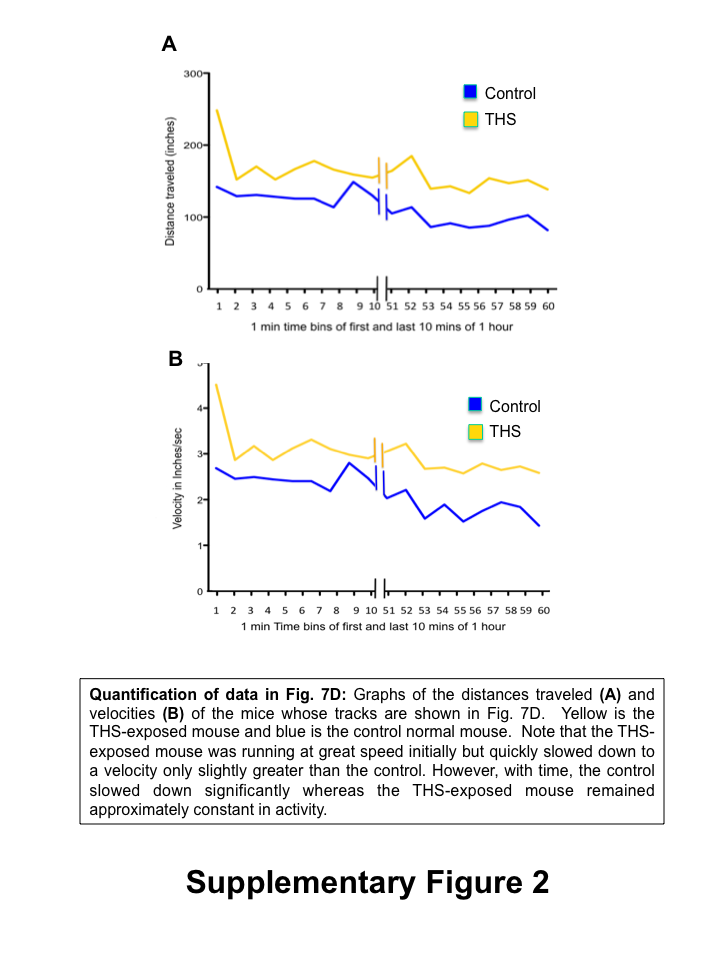

Supplement: Figure S2 — Quantification of data in Fig. 7D : Graphs of the distances traveled (A) and (B) velocities of the mice whose tracks are shown in Fig. 7D . Yellow is the THS exposed mouse and the blue is the control normal mouse. Note that the THS-exposed mouse was running at a great speed initially but quickly slowed down to a velocity only slightly greater than the control. However, with time, the control slowed down significantly whereas the THS-exposed mouse remained approximately constant in activity. (TIF) [file pone.0086391.s002.tif]
